# Catheter Interventions for Pulmonary Embolism: Mechanical Thrombectomy Versus Thrombolytics

**DOI:** 10.14797/mdcvj.1344

**Published:** 2024-05-16

**Authors:** Nicolas J. Mouawad

**Affiliations:** 1 McLaren Health System, Bay City, Michigan, US

**Keywords:** pulmonary embolism, PE, thrombolytics, mechanical thrombectomy

## Abstract

Pulmonary embolism is a debilitating and potentially life-threatening disease characterized by high mortality and long-term adverse outcomes. Traditional treatment options are fraught with serious bleeding risks and incomplete thrombus removal, necessitating the development of innovative treatment strategies. While new interventional approaches offer promising potential for improved outcomes with fewer serious complications, their rapid development and need for more comparative clinical evidence makes it challenging for physicians to select the optimal treatment for each patient among the many options. This review summarizes the current published clinical data for both traditional treatments and more recent interventional approaches indicated for pulmonary embolism. While published studies thus far suggest that these newer interventional devices offer safe and effective options, more data is needed to understand their impact relative to the standard of care. The studies in progress that are anticipated to provide needed evidence are reviewed here since they will be critical for helping physicians make informed treatment choices and potentially driving necessary guideline changes.

## Introduction

Pulmonary embolism (PE) remains a challenging disease, with rising prevalence and high mortality.^1^ Additionally, long-term adverse outcomes are common in survivors, with residual pulmonary vascular obstruction leading to diminished pulmonary function, known as post-PE syndrome (PPES), and chronic thromboembolic pulmonary hypertension (CTEPH).^2^ Mortality and PPES rates have changed little over several years,^3^ emphasizing the need for improved treatment modalities.

Treatment recommendations differ depending on PE severity. Several disease severity scores have been developed with varying use, with the most widely used being the Pulmonary Embolism Severity Index (PESI) and its simplified version (sPESI).^4,5^ The European Society of Cardiology (ESC) guidelines on classification of PE severity, which includes both PESI and sPESI, and risk of early death has become a well-established method for disease classification and resulting treatment guidance.^6^ Table 1 is a summary of the risk stratification classified by disease severity.

**Table 1:**
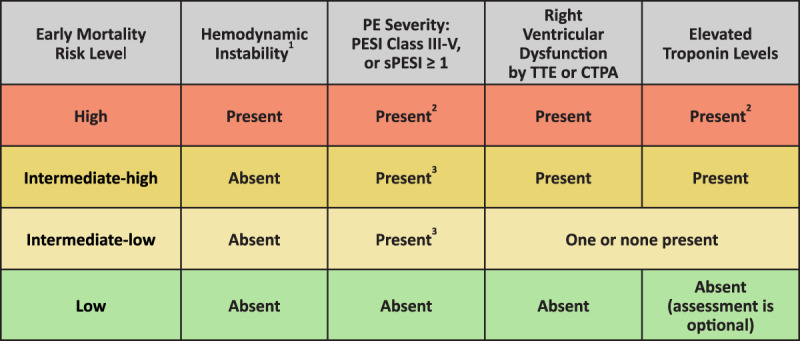
Pulmonary embolism severity classification and risk of death within 30 days. Adapted from Konstantinides et al.^6^ PE: pulmonary embolism; PESI: pulmonary embolism severity index; sPESI: simplified pulmonary embolism severity index; TTE: transthoracic echocardiogram; CTPA: computed tomography pulmonary angiogram; RV: right ventricular ^1^Hemodynamic instability is defined as the presence of one or more of the following: cardiac arrest, systolic blood pressure < 90 mm Hg or vasopressors required to achieve systolic blood pressure ≥ 90 mm Hg along with end-organ hypoperfusion, or persistent hypotension (systolic blood pressure < 90 mm Hg or a systolic blood pressure drop ≥ 40 mm Hg for > 15 minutes, not caused by new-onset arrhythmia, hypovolemia, or sepsis). ^2^Presence of hemodynamic instability and right ventricular dysfunction are sufficient for high-risk designation; sPESI/PESI score calculation and troponin measurements are not required in these cases. ^3^Patients with signs of RV dysfunction and/or elevated troponins should be classified as intermediate-high or intermediate-low risk even if a high sPESI/PESI score is absent.

Systemic thrombolytics (ST) are the guideline-recommended treatments for patients with high-risk (massive) PE, with surgical embolectomy or percutaneous catheter-directed treatment considered when thrombolytics have failed or are contraindicated.^6,7^ Despite this recommendation, only 30% of high-risk patients receive this treatment, likely due to bleeding risk concerns. Furthermore, only one in three patients receives any of these reperfusion therapies.^8^

For intermediate-risk (submassive) and low-risk patients, conservative medical management with anticoagulants (ACs) continues to be the guideline-driven standard of care, with rescue thrombolytics or other catheter-directed treatments suggested only when hemodynamic deterioration occurs while on ACs.^6^ The underuse of the recommended reperfusion therapies for high-risk patients along with the limited effectiveness of ACs in treating existing thrombotic obstruction may be contributing to the persistent mortality and PPES rates.

Recent studies also demonstrate that many intermediate-risk PE patients were experiencing cardiogenic shock despite their normotensive presentation,^9,10^ perhaps contributing to the unacceptable 90-day mortality rate of 12% in these patients.^11^ Arguably, these intermediate-risk patients on the precipice of hemodynamic deterioration may be better served with more aggressive interventional reperfusion therapy than ACs alone to rapidly relieve right ventricular (RV) strain and interrupt the death spiral seen in these decompensating patients.

New interventional reperfusion treatment modalities using pharmacological, mechanical, and/or aspiration methods are designed to reduce the bleeding risks that accompany ST as well as provide more rapid and effective thrombus removal.^12,13^ While new interventional approaches may provide optimal treatment options for many PE patients, their rapid development has outpaced published guidelines, leaving a potential gap between guideline recommendations and effective real-world treatment options. This review describes and compares endovascular treatment options currently in use that are indicated for PE and summarizes the published clinical evidence and several ongoing clinical studies that are anticipated to provide much-needed clinical data to evaluate the safety and effectiveness of these new treatment modalities.

## Limitations of Anticoagulation Therapy and Systemic Thrombolytics

Anticoagulation has been the mainstay treatment for venous thromboembolism. ACs have no intrinsic ability to break down existing thrombus, and thus the treatment relies on the body’s limited intrinsic thrombolytic ability while the anticoagulants prevent propagation of additional thrombi. To more effectively eliminate thrombotic obstruction, the use of STs such as tenecteplase and alteplase was adopted due to their ability to degrade fibrin, facilitating the breakdown of existing fibrin-rich thrombus. For several years, intravenous administration of ST was the main treatment choice to reduce PE thrombus load, which was shown in the Pulmonary Embolism Thrombolysis (PEITHO) randomized controlled trial (RCT) to significantly reduce a composite end point of death or decompensation at 7 days in intermediate-risk PE patients compared to AC alone.^14^ Evidence for the effectiveness of thrombolytics in high-risk patients, however, is limited.

A meta-analysis examining studies that included high-risk patients showed a mortality benefit of thrombolytics over AC, but the significance was lost after exclusion of the one study that enrolled only high-risk patients.^15^ This small RCT (N = 8) was halted early following 100% mortality in the first four AC patients and 0% mortality in ST patients.^16^ However, the positive outcomes from thrombolytic treatment were achieved at the cost of a several-fold increase in major bleeding, including intracranial hemorrhage.^14,15,17^

Furthermore, no difference in long-term incidence of CTEPH was found following systemic thrombolytic treatment compared to AC alone in a PEITHO substudy.^18^ In addition, ST-treated patients need to be monitored in intensive care units (ICUs) due to the high risk of bleeding, thus requiring significant hospital resources and prolonged hospital stays. These outcomes remained unchanged despite the use of modern thrombolytic agents and improved periprocedural patient management. In light of these limitations, interest in and adoption of interventional catheter-based therapies has surged due to the reduced bleeding risk and improved long-term outcomes.

## Catheter-directed Thrombolytics and Mechanical/aspiration Thrombectomy

Two categories of catheter-based treatment options have developed in recent years: catheter-directed thrombolysis and lytic-free mechanical thrombectomy. Each method offers specific advantages and drawbacks, with varying amounts of peer-reviewed published clinical evidence. It is important for physicians to consider their own level of expertise with each therapy, their patients’ specific needs, and the risk-benefit profile for each individual case when choosing among these options.

### Catheter-directed Thrombolysis

To overcome the bleeding complications associated with ST, catheter-based localized thrombolytic delivery at lower doses than systemic thrombolytic regimens was developed; this entailed catheters being maneuvered into the pulmonary arteries to infuse thrombolytics directly to the obstructing thrombus. Infusion was typically implemented through a side-hole catheter such as the Uni-Fuse catheter (AngioDynamics) or Cragg-McNamara catheter (Medtronic), with either one catheter introduced into one femoral vein or two catheters introduced with one in each femoral vein. The choice of thrombolytic drug, dose, and time of infusion varied, with no widely accepted protocol.

Two small RCTs were published evaluating catheter-directed thrombolysis (CDT) using the Cragg-McNamara catheter versus AC for intermediate- or intermediate-high-risk patients. In the study described in Kroupa et al., CDT significantly reduced RV/left ventricular (LV) ratio with no intracranial or life-threatening bleeding reported, but the study population was small (N = 23).^19^ The CANARY trial evaluated 85 randomized patients and found that fewer CDT patients experienced a 3-month composite outcome of death or RV/LV > 0.9 than AC patients (4.3% vs 17.3%; *P* = .048), with numerically but non-significantly higher bleeding events in the CDT arm.^20^ CDT for high-risk PE was the subject of a meta-analysis published in 2009 in which CDT appeared safer and more effective than the systemic approach.^21^ The authors recommended that catheter-directed thrombolysis be considered as a first-line therapy for acute, massive PE. However, recent work suggests that even a catheter-directed approach may be associated with more bleeding events than AC in intermediate-risk patients.^22^

A more recent addition to the catheter-based thrombolytic device group is the Bashir pharmacomechanical infusion catheter (Thrombolex). This device consists of an expandable basket with six nitinol infusion limbs designed to be placed in the thrombus, expanded to fragment the thrombus, and then disperse thrombolytics across a wider cross-section, thus potentially utilizing a lower thrombolytic dose. The RESCUE trial is a prospective multicenter single-arm study assessing the safety and effecacy of this device in intermediate-risk PE patients. Acute results in 109 patients showed statistically significant reduction in 48-hour RV/LV ratio (0.56 reduction, *P < .001*) and in pulmonary artery (PA) obstruction by Miller score (35.9% reduction, *P < .001*), with three patients (2.7%) experiencing procedure-related serious adverse events.^23^

### Ultrasound-accelerated Thrombolysis

To potentially further enhance the efficacy of CDT, ultrasound technology was incorporated into the CDT catheter to accelerate the lytic process by thinning and separating fibrin strands within the thrombus, which in vitro studies had shown to be effective on non-Factor-XIII-crosslinked fibers.^24^ This led to the development of the EkoSonic catheter system (EKOS™, Boston Scientific). This ultrasound-assisted thrombolysis (USAT) approach was evaluated in two multicenter prospective studies, ULTIMA and SEATTLE-II.

ULTIMA was a randomized study of USAT using 10-mg to 20-mg recombinant tissue plasminogen activator (tPA) infused over 15 hours with AC versus AC alone in 59 subjects with intermediate-risk PE.^25^ USAT was found to promote significantly greater RV/LV reduction compared to AC at 24 hours, but the difference was not significant at 90 days, at which time AC patients showed similar RV/LV ratio as the USAT patients. There were no major bleeding events in either group (three minor bleeds in USAT and one in AC) and no intracranial bleeding was observed. However, the study authors noted, “The study was too small to draw firm conclusions about the clinical efficacy and safety of USAT in comparison to AC alone.”^25^

The SEATTLE-II single-arm prospective study evaluated 24 mg of tPA infused over 24 hours in one catheter or over 12 hours in two catheters in 150 subjects with intermediate- and high-risk PE.^26^ Postprocedure mean pulmonary artery pressure and modified Miller Index score for pulmonary obstruction showed significant improvements, as did 48-hour RV/LV ratio, with 15 patients experiencing major bleeding events over 30 days (1 GUSTO severe bleed) and no intracranial hemorrhage.^26^ Further refinement of the lytic dose and infusion duration using EKOS was conducted in the OPTALYSE PE trial, which included four study arms with tPA doses ranging from 4 mg to 12 mg per lung and infusion duration from 2 to 6 hours.^27^ Long-term outcomes from this study were also assessed.^28^ These studies concluded that CDT with tPA was safe and effective in the treatment of intermediate-risk PE, at least with respect to reductions in RV/LV ratio, and they suggested that lower doses and infusion durations were associated with significant RV/LV improvements and clot burden reduction at 48 hours.

These conclusions, however, have not been without controversy. A 2017 review of 24 studies and 700 subjects found the rate of total bleeding events for USAT and conventional CDT to be 12% and 10%, respectively, with major bleeding rates of 4% and 10%, respectively. While the review documented a trend toward improved survival with USAT (mortality rates of 4% vs 9% in the USAT and CDT subjects, respectively), the authors concluded that clinical evidence did not show USAT superiority over CDT.^29^ A more recent prospective RCT (SUNSET PE) comparing USAT to standard CDT in 81 intermediate-risk PE patients demonstrated no benefit of USAT compared to standard CDT in thrombus reduction, with significantly greater reduction in RV/LV ratio seen in the standard CDT group in spite of similar thrombolytic dose and infusion times. The two major and three minor bleeding events in the study all occurred in the USAT group, as did the one study death. The authors concluded that incorporation of ultrasound technology into CDT treatment may not confer additional benefit.^30^

When considering the published results summarized above, it is important to keep in mind that these studies were performed on the subset of PE patients who have no contraindications to thrombolytics due to bleeding risks. Several clinical factors are considered either absolute (eg, history of intracranial bleeding, active bleeding) or relative contraindications (eg, recent surgery, pregnancy, older age),^6^ thus limiting the patient population amenable to these treatments. Further limiting the patient population is thrombolytics’ mechanism of action against fibrin. While acute venous thrombi are fibrin-rich, thus providing an excellent target for thrombolytic drugs, thrombi undergo modifications over time, with fibrin diminishing as it is replaced with collagen-rich fibrotic material. Within 2 weeks, collagen comprises 50% to 80% of the thrombus, with further fibrotic progression to wall-adherent, scar-like post-thrombotic material, which is much less amenable to thrombolytic degradation.^31^ Furthermore, there is often a discrepancy between the actual age of thrombus and patient-reported symptom duration, with over 90% of patients with “old” thrombi as designated by magnetic resonance imaging analysis reporting only acute or subacute symptom duration.^32^ Thus, the effectiveness of CDT demonstrated in clinical studies of patients without thrombolytic contraindications and with only acute thrombus may not be applicable to the larger real-world PE patient population.

### Mechanical Thrombectomy

The continued pursuit of improved thrombus removal methods for a broader PE patient population—to provide more immediate hemodynamic relief, leaving minimal residual thrombus without the bleeding risk or requisite ICU stay—led to the development of lytic-free mechanical thrombectomy techniques. These catheter-based approaches involve the mechanical removal of thrombus from the pulmonary vasculature either through aspiration (suction) or through entrapment of the thrombus using mechanical tools (extirpation). Interest in this purely mechanical approach has increased due to its rapid treatment versus the relatively slow infusion rate of thrombolytic treatment, and without the inherent risk of bleeding complications seen with pharmacologic thrombolysis. In addition, percutaneous thrombectomy can be performed in the catheterization lab without the need for an ICU stay required during thrombolytic infusion. The lack of ICU requirement became particularly attractive during the COVID-19 pandemic with ICU bed shortages.

Mechanical thrombectomy also provides a much-needed treatment option for the up-to-50% of PE patients who are contraindicated for thrombolytics. This treatment option also may be more effective at removing older thrombus, which are not amenable to thrombolytic drugs. Furthermore, the ability to effectively remove thrombus regardless of its age may have a beneficial impact on long-term outcomes by reducing residual thrombus and the subsequent development of post-PE syndrome and/or CTEPH and its debilitating impact on functional status and quality of life.^33,34,35^ Multiple mechanical thrombectomy devices with slightly differing approaches have been developed. This review focuses on those mechanical thrombectomy devices currently indicated for treatment of PE, which include the Indigo™ Aspiration System (Penumbra) and the FlowTriever System™ (Inari Medical).

The Indigo System consists of catheters available in up to 16F diameter connected to an aspiration engine with computer-assisted technology to control aspiration, and reduce potential blood loss, based on the detected presence of thrombus. The EXTRACT-PE trial was a single-arm prospective study that enrolled 119 acute PE patients at 22 sites in the United States (US) from Nov 2017 to March 2019 to evaluate RV/LV ratio and adverse events at 48 hours. Patients achieved a mean RV/LV ratio reduction of 0.43 (1.47 to 1.04; *P* < .001), with a 1.7% major adverse event rate at 48 hours, including one patient with a major bleed and another with device-related hemoptysis, major bleed, deterioration, and death. Two patients (1.7%) received adjunctive thrombolytics, and 61% of patients required an ICU stay.^36^ Two additional studies are currently enrolling: the STRIKE-PE trial will enroll up to 600 patients to evaluate long-term safety and outcomes following Indigo treatment, while the STORM-PE RCT will compare outcomes in PE patients treated with Indigo versus AC alone. Additional nonindustry reports of single- and multicenter studies using the Indigo system to treat PE have been published, each demonstrating significant improvements in pulmonary function with acceptable safety outcomes.^37,38,39^

**Figure 1:**
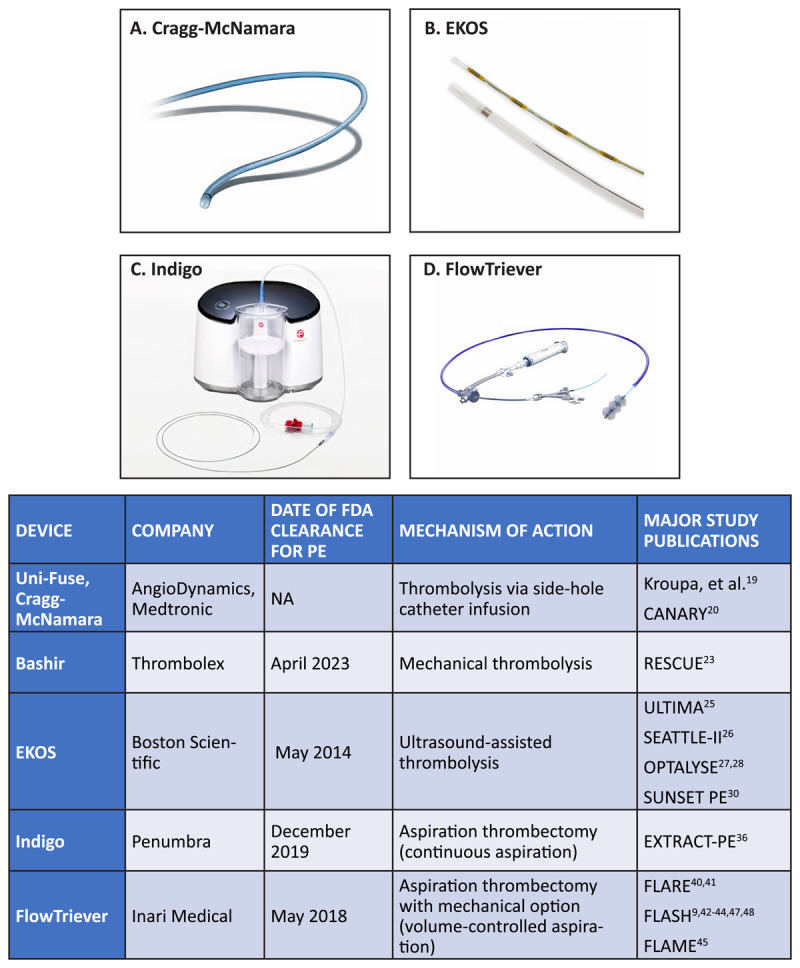
Interventional devices for pulmonary embolism treatment. Copyright 2024; reprinted with permission from Medtronic, Boston Scientific, Penumbra, and Inari Medical. FDA: Food and Drug Administration; PE: pulmonary embolism

The FlowTriever System (Inari Medical) is composed of two main components: the Triever aspiration catheters available in several configurations with 16F, 20F, and 24F diameters, along with T16C and T20C curved catheters, and the FlowTriever catheters comprised of nitinol disks of different sizes designed to be deployed and expanded at the thrombus to macerate and deliver the thrombus into the Triever catheter for removal by aspiration. The Triever catheters are often used as the main approach to thrombus removal, with FlowTriever disks employed for more intractable thrombus cases. Aspiration is created manually with a 60-mL custom large-bore syringe connected to a catheter side port, which limits blood loss to 60 mL per aspiration. Due to the potential of blood loss that occurs with aspiration, the FlowSaver™ Blood Return System was also developed, which is a separate product for filtering and reintroducing blood aspirated by the FlowTriever System. Figure 1 shows a list of endovascular options for PE treatment.

The FlowTriever System was first evaluated in the prospective single-arm FLARE study, which enrolled 106 intermediate-risk PE patients from April 2016 to October 2017 to evaluate RV/LV ratio and major adverse events at 48 hours, with additional follow-up to 30 days. Two patients received adjunctive thrombolytics. The mean reduction in RV/LV ratio from baseline to 48 hours was 0.38 (25.1%, *P* < .001), and there were four patients with major adverse events involving clinical deterioration (3.8%). The study met its success criteria, resulting in FlowTriever being the first mechanical thrombectomy system to be indicated for PE treatment.^40^ A substudy of FLARE patients diagnosed in emergency room departments also demonstrated significant improvement in RV/LV ratio with low complication rates.^41^

Following multiple design changes, the next-generation FlowTriever device was evaluated in the FLASH registry, a prospective single-arm study that enrolled 800 patients across 50 US sites that included both high- (8.0%) and intermediate-risk (92.0%) patients. Patient enrollment of up to 200 European Union patients is currently ongoing. Acute results have demonstrated significant reductions in hemodynamic parameters, including a reduction of mean pulmonary artery pressure from 32.6 mm Hg preprocedure to 24.9 mm Hg postprocedure (*P* < .001). Additional effectiveness outcomes included significant reduction in several RV echocardiographic parameters and significant reduction in the modified Medical Research Council dyspnea score from a median of 2.7 at baseline to 1.1 at 48 hours. Major adverse event and 48-hour mortality rates were low at 1.8% and 0.3%, respectively.^42,43^

**Figure 2:**
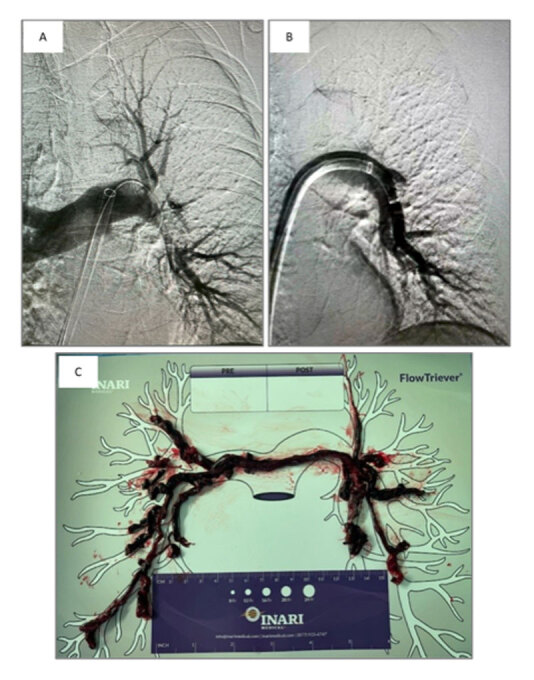
FlowTriever case examples. (A) Pre-thrombectomy angiogram of the left pulmonary artery with thrombus. (B) Post-thrombectomy angiogram of the same left pulmonary artery showing absence of thrombus and return of blood flow. (C) Successful thrombus extraction of patient presenting with massive saddle pulmonary embolism.

Importantly, these improvements seen at 48 hours were maintained out to 6 months, with median dyspnea score of 0.0 at 30 days and 6 months, and median RV/LV ratio of 0.80 at 6 months.^44^ Additional 6-month patient-centric outcomes were measured, including a median 6-minute walk test distance of 398 m, and a median PE-specific quality of life measurement (PEmb-QoL) score of 4.85 (out of 100, 0 being best). Mortality remained low with a 6-month all-cause mortality of 4.8%, and site-reported chronic thromboembolic disease and CTEPH rates were 1.9% and 1.0%, respectively, at 6 months.^44^ Case examples of FlowTriever use are shown in Figure 2.

The safety and effectiveness of FlowTriever thrombectomy has also been evaluated in a high-risk patient population in the FLAME study.^45^ The study, designed according to recommendations in the 2019 American Heart Association Scientific Statement for acute PE,^46^ was a prospective multicenter nonrandomized parallel-group observational study with high-risk PE patients enrolled into either a FlowTriever arm (n = 53) or a context arm (other non-FlowTriever treatments, n = 61; 68.6% were systemic thrombolytics, 23.0% were AC alone). Due to the nonrandomized design, outcomes in the two arms were not directly compared. Outcomes in the FlowTriever arm were compared to a prespecified performance goal based on historical data. The primary end point was an in-hospital composite of all-cause mortality, bailout to alternate thrombus removal therapy, clinical deterioration, and major bleeding. The performance goals were 32% for the primary composite end point, and 28.5% for all-cause in-hospital mortality. In comparison, the primary composite end point was reached in 17.0% of FlowTriever arm patients, with an all-cause mortality rate of 1.9%.

Results for the context arm patients showed that 63.9% reached the primary composite end point with an all-cause mortality of 29.5%.^45^ The high-risk population subset in FLASH also has been evaluated and demonstrated positive outcomes, with zero mortalities seen in the 63 high-risk patients at 48 hours and significant reduction in RV/LV ratio from 1.5 at baseline to 1.1 at 48 hours and 0.9 at 30 days.^47^ Analysis of a subset of FLASH patients in whom intravascular ultrasound was used pre- and post-thrombectomy^48^ as well as several non-industry single-center studies of FlowTriever have also been published, including single-center and multicenter evaluations,^49,50,51,52,53^ showing comparable safety and effectiveness outcomes as demonstrated by the FLASH registry data. Table 2 summarizes the risks and benefits of PE treatments discussed in this review.

**Table 2:**
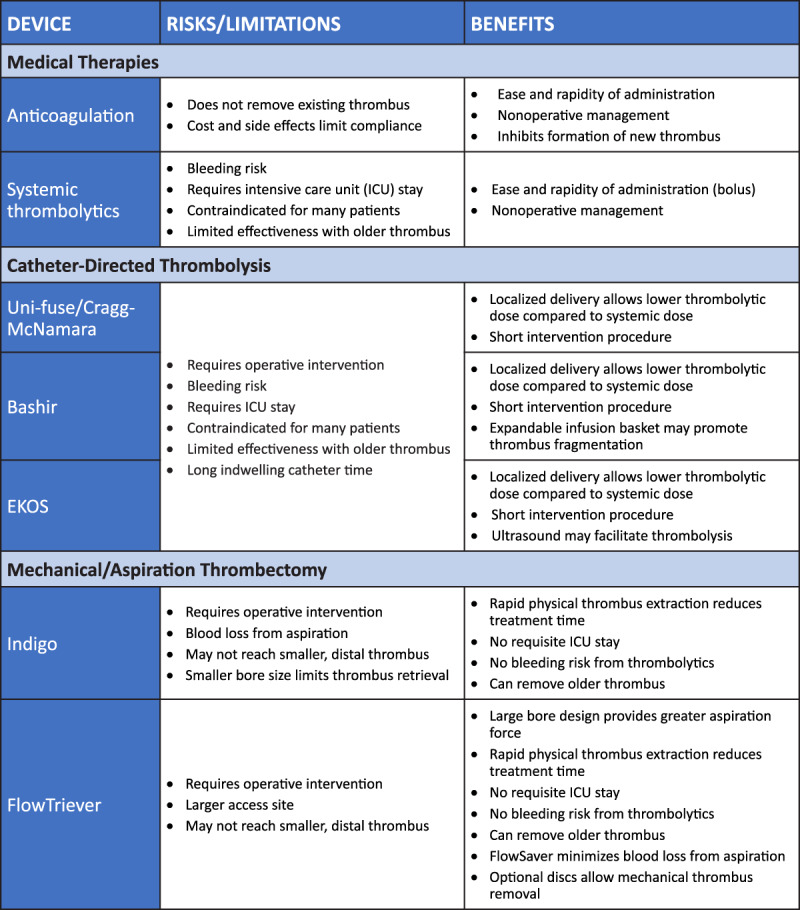
Risks and benefits of current treatments for pulmonary embolism.

## Forthcoming Randomized Controlled Trials

While there are no published results of randomized trials comparing mechanical thrombectomy to either AC or CDT, several RCTs are currently enrolling with expected outcomes available in late 2024. The PEERLESS trial (NCT05111613) will randomize 550 intermediate-risk patients 1:1 to a FlowTriever mechanical thrombectomy arm and a CDT arm, with 30-day follow-up,^54^ while PEERLESS II (NCT06055920)^55^ will randomize up to 1,200 intermediate-risk patients 1:1 to a FlowTriever mechanical thrombectomy arm and an AC-only arm, with 90-day follow-up. STORM-PE (NCT05684796)^56^ will randomize 100 intermediate-risk patients 1:1 to an Indigo mechanical thrombectomy arm and an AC-only arm with 90-day follow-up. A fourth RCT, HI-PEITHO (NCT04790370), will randomize up to 544 patients to either an EKOS USAT arm or an AC-only arm with 12-month follow-up.^57^ A fifth RCT, and the only currently-enrolling study that is non-industry sponsored, is PE-TRACT (NCT05591118), which will compare an AC-only arm to a catheter-directed therapy arm that can include either MT or CDT devices.^58^ Up to 500 intermediate-risk patients will be enrolled with a 12-month follow-up. While it may be several years before results from these RCTs are reported, this wealth of data, once available, should help fill the evidence gap and drive a much-needed consensus in PE treatment options and recommendations. Table 3 shows a timeline of relevant PE treatment publications and details.

**Table 3:**
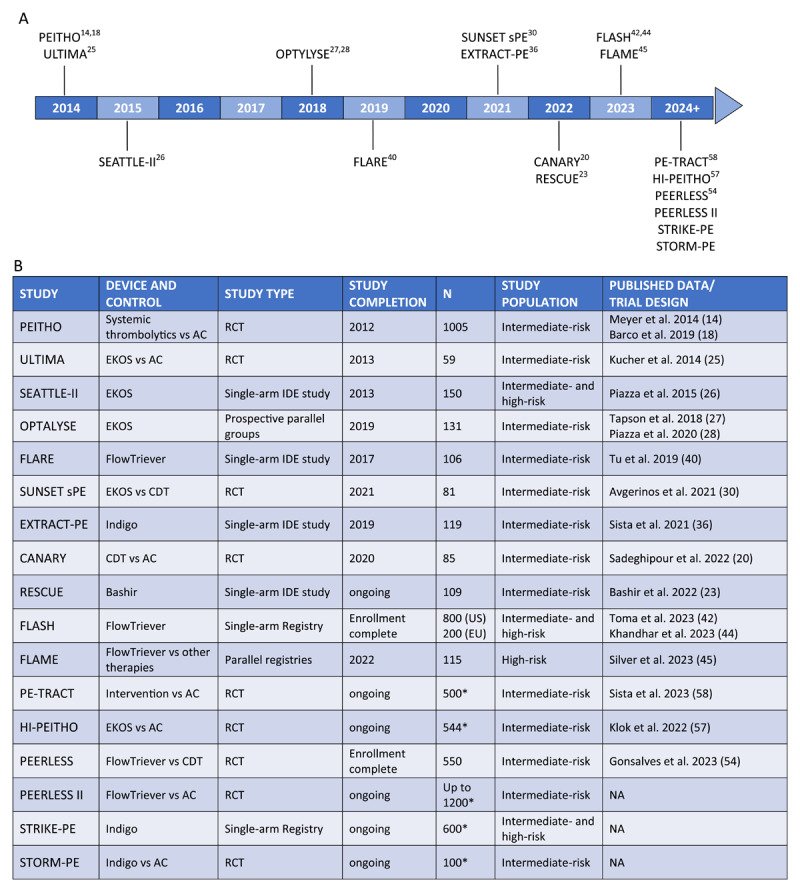
**(A)** Timeline of relevant pulmonary embolism treatment study publications. **(B)** Table with study details. AC: anticoagulation; RCT: randomized controlled trial; IDE: investigational device exemption; CDT: catheter-directed thrombolysis; NA: not applicable * Target enrollment; currently enrolling.

## Conclusion

While the safe and effective treatment of PE and other venous thromboembolism diseases has lagged behind other vascular obstruction disease states (such as myocardial infarction and stroke), the rapid pace of recent endovascular innovations in PE treatment options and a plethora of ongoing clinical studies, including RCTs, hold great promise for the advancement of PE patient care. The multiple device options currently available and in development offer a range of treatment modalities that allow physicians to match the optimal treatment to each patient’s needs, although discerning the optimal choice among the many options may be difficult. The clinical data generated thus far suggests that mechanical thrombectomy offers a strong, safe, and effective treatment approach without the bleeding risks and limited thrombus age efficacy of thrombolytics. However, head-to-head comparative data are needed. The generation and publication of high-quality clinical evidence from the ongoing RCTs will be critical for physicians to make informed choices for their patients and to bring guideline recommendations in line with the current treatment technologies.

## Key Points

- The development of interventional thrombus-removal techniques has outpaced current published guideline recommendations for the treatment of pulmonary embolism (PE).
- Recent published data evaluating the safety and effectiveness of both catheter-directed thrombolysis and mechanical thrombectomy suggest that the use of catheter-based interventions may be warranted for more PE patients than what guidelines recommend.
- The optimal patient selection, potential effectiveness, and possible complications differ between thrombolytic treatment and mechanical thrombectomy, necessitating a good understanding of the attributes of each device type in order to choose the best option for each patient.
- Both catheter-directed treatment approaches have limitations. The bleeding risk as well as the reduced effectiveness against subchronic and chronic thrombus seen with catheter-directed thrombolytic treatment limit the patient population that can be treated safely and effectively with this option. Mechanical thrombectomy requires the ability to anatomically navigate catheters to the thrombus to physically extract it, which is not always possible depending on vascular anatomy and thrombus location, particularly for distal thrombus. Aspiration techniques that lack a blood return option may lead to significant blood loss and the need for blood transfusions.
- Ongoing randomized controlled trials are expected to provide robust evidence to further support recommendations regarding specific treatment modalities.
